# Mixed phenotype acute leukemia, the dissection of an enigmatic disease in the era of novel therapies

**DOI:** 10.3389/fped.2025.1715087

**Published:** 2025-12-16

**Authors:** Magdalena Karasek, Izabela Kubiszewska, Marta Sobas

**Affiliations:** 1 Clinical Department of Hematology, Cell Therapies and Internal Diseases, Wroclaw Medical University, Wroclaw, Poland; 2 Department of Immunology, Nicolaus Copernicus University in Torun, Collegium Medicum in Bydgoszcz, Bydgoszcz, Poland; 3 Department of Laboratory Diagnostics, Jan Biziel University Hospital No. 2 in Bydgoszcz, Bydgoszcz, Poland; 4 Department of Hematology, Collegium Medicum in Bydgoszcz, Nicolaus Copernicus University in Toruń, Bydgoszcz, Poland

**Keywords:** mixed-phenotype acute leukemia, outcome, genetic variability, targeted therapy, acute leukemia of ambiguous lineage

## Abstract

**Background:**

Mixed-phenotype acute leukemia (MPAL) is a rare and heterogeneous subtype of acute leukemia, associated with unfavorable outcomes. MPAL is defined by the presence of more than 20% blasts and bi- or trilineage assignment based on strong immunophenotypic markers, with specific subcategories characterized by *BCR::ABL1*, *KMT2A*, *ZNF384*, or *BCL11B* rearrangements. This review aims to summarize current knowledge and challenges in the diagnosis and management of MPAL.

**Methods:**

Data were synthesized primarily from meta-analyses and original studies, with a particular emphasis on the roles of immunophenotyping, cytogenetics, and novel targeted therapies from 1985 to the present.

**Results:**

MPAL accounts for 1%–5% of acute leukemias, with B/myeloid (59%) and T/myeloid (35%) subtypes being the most prevalent. Cytogenetic abnormalities are identified in up to 90% of cases, predominantly complex karyotypes. Molecular investigations have identified frequent mutations in genes such as *RUNX1*, *DNMT3A*, *IDH1/2*, *NOTCH1*, and *FLT3*, particularly enriched in T/myeloid MPAL. Adverse prognostic factors include *KMT2Ar*, elevated leukocyte counts, extramedullary disease, and bilineage disease biology. Generally, the prognosis for adults is poorer than for the pediatric population. No standardized treatment strategy has been established. Retrospective analyses indicate superior complete remission rates and overall survival with ALL-based regimens, and allogeneic hematopoietic stem cell transplantation remains crucial for improving survival. Recently, hybrid regimens such as FLAG-IDA and CLAG-M have demonstrated promising efficacy with acceptable toxicity. Targeted therapies are emerging options, although lineage switch under selective therapeutic pressure remains a concern.

**Conclusions:**

MPAL remains a significant challenge in diagnosis and treatment. Advances in molecular characterization have enhanced classification techniques and have the potential to inform personalized treatment strategies. Considering the rarity and heterogeneity of MPAL, extensive prospective multicenter trials are imperative to develop evidence-based therapeutic protocols.

## Introduction

Acute leukemia of ambiguous lineage (ALAL) comprises rare subtypes of leukemia that do not meet criteria for classification within a single hematopoietic lineage. ALAL encompasses acute leukemias lacking defining lineage features (acute undifferentiated leukemia, AUL) and those characterized by immunophenotypic markers of multiple lineages (mixed-phenotype acute leukemia, MPAL) (1).

The clinical presentation of MPAL resembles that of other acute leukemias (2–8); however, hepatosplenomegaly, lymphadenopathy, and central nervous system (CNS) involvement are more commonly observed at the time of diagnosis (5, 6).

MPAL is associated with an unfavorable prognosis and inferior treatment response compared with acute myeloid leukemia (AML) and acute lymphoblastic leukemia (ALL). Therapeutic outcomes in MPAL are influenced by patient age (with a more favorable prognosis in pediatric populations) and genetic alterations (2–4, 9, 10). Currently, no standardized treatment protocol has been established for MPAL, owing to its rarity and the lack of prospective clinical trials. Based on retrospective reports conducted in heterogeneous patient groups, longer survival has been achieved with ALL-based chemotherapy regimens (mainly pediatric protocols), mostly consolidated with allogeneic hematopoietic stem cell transplantation (alloHSCT) (2–4, 10, 11). Increasing interest has been directed toward hybrid protocols, which demonstrate cytotoxic activity against blasts of both myeloid and lymphoid origin. Targeted therapy also represents a promising therapeutic strategy (12–14).

This review aims to synthesize current knowledge of the biological foundations of MPAL, elucidate the major diagnostic challenges, and highlight emerging avenues for personalized therapeutic strategies to improve overall survival. Owing to the rarity of this leukemia, the review includes evidence and recommendations relevant to both pediatric and adult patient populations.

## Methods

A comprehensive literature search was conducted to identify publications relevant to MPAL. Data were retrieved from major biomedical databases, including PubMed, EBSCO, and Web of Science. The search strategy incorporated the following key terms and their combinations: “Mixed-Phenotype Acute Leukemia”, “MPAL”, “Acute Leukemia of Ambiguous Lineage”, and “ALAL”. The search encompassed articles published from 1985 to the present, restricted to English-language publications, and covered both adult and pediatric patient populations.

Publications were systematically screened and prioritized based on their level of evidence. Meta-analyses were given precedence, succeeded by systematic and narrative reviews, prospective and retrospective multicenter studies, and ultimately, single-center experiences and case reports. References cited within pertinent reviews were also meticulously examined to identify further eligible sources.

Considering the rapid progress in molecular diagnostic techniques, the revision of classification criteria, and the emergence of targeted therapeutic approaches, particular emphasis was placed on the most recent and methodologically robust studies.

### Classification and diagnostic criteria of MPAL

Regarding the recent WHO and ICC classifications ALAL and MPAL are classified together owing to their clinical, immunophenotypic, and molecular similarities (1, 15). A significant challenge in this classification lies in the fact that recurrent mutations alone are insufficient to elucidate the phenotypic heterogeneity observed in these leukemias fully. The lineages are determined based on the expression of cytoplasmic and surface antigens on blast cells (1, 15, 16).

**Figure 1:**
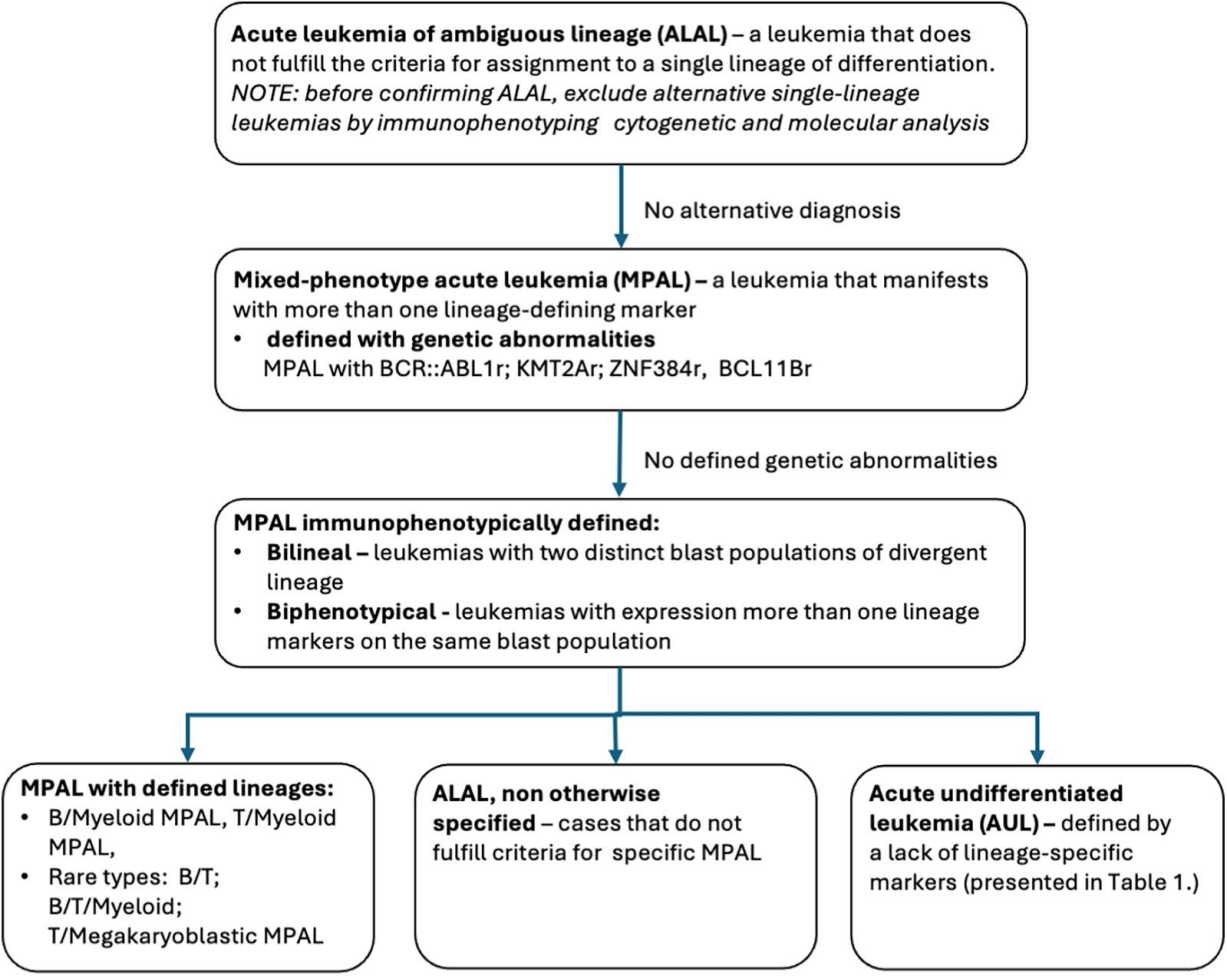
Diagnostic algorithm of ALAL/MPAL.

To facilitate diagnosis, the European Group for the Immunological Characterization of Leukemias (EGIL) developed a scoring system in 1995 (16), which was later revised by the World Health Organization (WHO). Subsequent changes in the diagnostic criteria for MPAL are summarized in Table 1. The current diagnosis of ALAL requires the presence of >20% blasts of undefined lineage in the bone marrow, only after excluding well-defined AML subtypes (1). In MPAL, the cases are subsequently categorized into those exhibiting specific genetic abnormalities and those characterized exclusively by immunophenotypic features (B/myeloid, T/myeloid, B/T/myeloid, T/megakaryoblastic subtypes) (Figure 1). In the current WHO/ICC classification, the category with specific genetic abnormalities includes:

- MPAL with *BCR::ABL1* fusion,
- MPAL with *KMT2A* rearrangement (*KMT2Ar*),
- two recently recognized subtypes: MPAL with *ZNF384* rearrangement (*ZNF384r*) and MPAL with *BCL11B* rearrangement (*BCL11Br*) (1).

**Table 1:** The comparison of the current diagnostic criteria.

EGIL classification			
	2 points	1 point	0,5 point
Myeloid lineage	Myeloperoxidase (MPO) Lysozyme	CD117, CD33, CD13, CD65a	CD14, CD15, CD64
T-lymphoid lineage	CD3 (cytoplasmic or surface), anti-TCR alfa/beta, anti-TCR gamma, delta	CD2, CD10, CD5, CD8	TdT, CD1a, CD7
B-lymphoid lineage	Cd79A, cytIgM, cytCD22	CD19, CD20, CD10	TdT, CD24
To be considered positive, a marker must be expressed on ≥20% of blasts; the exceptions are MPO, CD3, and CD79a, for which, due to their high specificity, a threshold of ≥10% of blasts is sufficient. Lineage assignment is established when a score of more than 2 points is obtained.			
WHO 2022 Classification			
Myeloid lineage: MPO: fluorescence intensity exceeding 50% of that observed in mature neutrophils, or monocytic differentiation markers: ≥2 of the following: nonspecific esterase, CD11c, CD14, CD64, lysozyme.			
T-lymphoid lineage: CD3 (cytoplasmic or surface)¹: fluorescence intensity exceeding 50% of that observed in mature T lymphocytes, or positive immunohistochemistry with an antibody not directed against the *ζ*-chain.			
B-lymphoid lineage: If CD19 is strongly expressed²: at least one additional strongly expressed marker (CD10, CD22, CD79a⁴). If CD19 is weakly expressed³: at least two additional strongly expressed markers (CD10, CD22, CD79a⁴).			
¹ Using anti-CD3 antibody against the *ε*-chain, ² CD19 fluorescence intensity >50% of that observed in B-cell progenitors by flow cytometry, ³ CD19 fluorescence intensity ≤50% of that observed in B-cell progenitors by flow cytometry, ⁴ If T-lineage involvement is also suspected, CD79a cannot be used.			
Lineage assignment criteria apply only to cases in which MPAL is under consideration and are not universally applicable to the diagnosis of ALL or AML.			
MPAL should not be diagnosed in the setting of chronic myeloid leukemia in blast crisis, AML with dysplastic changes, therapy-related AML, or when *t*(8;21), *t*(15;17), or inv (16) is present. By contrast, NPM1 or CEBPA mutations do not preclude the diagnosis of MPAL.			
In cases with two distinct blast populations (bilineal leukemia), it is not necessary to fulfil the above criteria. Instead, each blast population should independently meet the diagnostic definition of B-, T-, or myeloid-lineage leukemia.			
MPAL diagnosis may not be appropriate in cases with typical B-ALL markers and uniform lymphoid antigen expression in a single blast population, with weak MPO expression as the only evidence of myeloid differentiation. Such cases should not be classified as MPAL.			
In challenging or ambiguous cases, the heterogeneity of antigen expression should be carefully assessed (e.g., populations with strong lymphoid marker expression often exhibit lower myeloid marker expression and vice versa). Heterogeneous antigen expression is a common feature of MPAL.			

The diagnosis of MPAL is predicated on the immunophenotypic characteristics of the blasts analyzed through flow cytometry. Verification of the heterogeneous origin of blasts is particularly crucial in instances of a homogeneous blast population lacking distinct morphological features. According to the current WHO guidelines, the most lineage-specific membrane and cytoplasmic receptors are employed in the cytometric diagnosis of MPAL (Table 1) (1). In MPAL, both the presence of the receptor and its density on blast cells are of significance. A high receptor density should be defined as that observed on normal cell lines, present in at least 50% of blast cells. MPAL can be accurately diagnosed using standard testing protocols for acute leukemias, such as the ALOT (Acute Leukemia Orientation Tube) and the AML/MDS diagnostic panel (Table 2).

**Table 2:** The comparison of cytometric diagnostic criteria between T/myeloid MPAL and early T-cell precursor ALL (ETP-ALL).

Cell markers	**T/myeloid MPAL**	**ETP-ALL**
MPO expression	MPO (+)	MPO (-).
Myeloid/stem cell markers	CD13, CD33, CD34, CD117.	CD13, CD33, CD34, CD117
Immature T-cell markers	cytCD3, CD7	cytCD3, CD7
Additional Markers	positive expression: CD2 and HLA-DR or monocytic markers (CD11c, CD14, lysozyme).	negative expression: CD5, CD1a, CD8,
		positive expression (some cases): CD2 and CD4.

Evaluation of myeloperoxidase (MPO) expression is integral, especially in differentiating minimally differentiated AML, T/myeloid MPAL, and early T-cell precursor ALL (ETP-ALL) as all of these entities may express certain myeloid markers (Table 3) (1).

**Table 3:** Antibody panel for immunophenotyping of AML (euroFlow).

Acute Leukemia Orientation *Tube* (ALOT)								
1	cytMPO	cytCD79A	CD34	CD19	CD7	CD3	cytCD3	CD45
AML/MDS flow cytometry panel								
1	CD16	CD13	CD34	CD117	CD11b	CD10	HLA-DR	CD45
2	CD35	CD64	CD34	CD117	CD300e	CD14	HLA-DR	CD45
3	CD36	CD105	CD34	CD117	CD33	CD71	HLA-DR	CD45
4	cytTdT	CD56	CD34	CD117	CD7	CD19	HLA-DR	CD45
5	CD15	NG2	CD34	CD117	CD22	CD38	HLA-DR	CD45
6	CD42a/CD61	CD203c	CD34	CD117	CD123	CD4	HLA-DR	CD45
7	CD41a	CD25	CD34	CD117	CD42b	CD9	HLA-DR	CD45

Although the WHO has not specified a definitive cut-off for MPO, various authors recommend a 3% threshold for MPO positivity by immunohistochemistry (IHC) and 10% by flow cytometry (FCM) (16, 17). Comparative studies have demonstrated higher sensitivity of IHC relative to FCM and cytochemistry techniques (18, 19). To enhance MPO detection sensitivity via FCM, some researchers suggest lowering the cut-off point to 5.4% (20). An additional challenge in the flow cytometric assessment of MPO is the potential for non-specific antibody binding, necessitating meticulous selection of antibodies for this method. In cases with borderline results or technical uncertainties, IHC or cytochemical testing is recommended. Caution is warranted when blast cells exhibiting lymphocytic lineage antigens also express MPO without other myeloid receptors. To distinguish MPAL from ALL (cases with low MPO expression), MPO assessment should be based on cytoplasmic density relative to control cells (neutrophils). According to the 2022 WHO guidelines, MPO expression is classified as low or absent if less than 50% of blast cells display MPO intensity comparable to or brighter than mature neutrophils, and as high if 50% or more of blast cells exhibit MPO intensity equivalent to that of mature neutrophils (1). The first scenario suggests an ALL with isolated expression of MPO (isoMPO) diagnosis, whereas the second indicates MPAL.

Recently, Weinberg et al. demonstrated that MPO as the only myeloid-defining marker is crucial to distinguish B-cell precursor ALL (BCR-ALL) from B/myeloid MPAL with isoMPO, To emphasize, B/myeloid MPAL with isoMPO did not differ cytogenetically from other subtypes of MPAL (21).

Emerging research indicates that immature leukemias and MPAL subtypes, which share similar genetic alterations, tend to overlap in their immunophenotypic profiles, thereby largely obscuring the artificial distinctions between these entities. Nevertheless, further studies are required, it appears that genetic alterations, rather than phenotypic expression, may represent a more effective basis for classifying these leukemias in the future (22, 23).

### Epidemiology

ALAL accounts for approximately 2%–3% of all acute leukemias, with the vast majority classified as MPAL (9). The incidence exhibits a slight male predominance: 0.45 in men compared to 0.26 in women per 1,000,000 inhabitants annually. The most prevalent subtype is B/myeloid MPAL (59%–72%), followed by T/myeloid MPAL (21%–32%). Other subtypes are considerably less common (2, 9, 24).

### Pathogenesis

The biological origin of MPAL continues to be a subject of ongoing debate, reflecting the notable heterogeneity of its genetic, epigenetic, and phenotypic landscape. Two principal, not mutually exclusive, models have been proposed to elucidate the emergence of the mixed-lineage phenotype.

1. **Lineage promiscuity**—this theory proposes that when leukemic transformation occurs at the level of a multipotent hematopoietic progenitor cell, it retains the potential for both myeloid and lymphoid differentiation with a mostly stable genome (25–29).
2. **Lineage infidelity—**this concept suggests that subsequent genetic mutations or transcriptional disruptions interfere with standard lineage commitment, resulting in abnormal co-expression of markers that define specific lineages (30–33).

The landmark study conducted by Alexander et al. employed a multistep experiment to elucidate the molecular and cellular origins of MPAL). The authors analyzed both adult and pediatric cases (age range 1–70, median 23) MPAL cases, comparing their mutational and transcriptional profiles with those of AML, ALL), and normal hematopoietic progenitor cell populations. The findings were ultimately validated through a definitive experiment utilizing NSG-SGM3 mouse models. Through gene expression and methylation profiling, it was demonstrated that MPALs frequently originate from multipotent hematopoietic progenitors that retain both myeloid and lymphoid potential, thereby supporting the lineage promiscuity model. Furthermore, recurrent genetic alterations involving *RUNX1*, *WT1*, *PHF6*, and *ZEB2* were identified, indicating shared leukemogenic mechanisms with other forms of acute leukemia. Additionally, decreased methylation levels of PAX5, CXCR4, and RAG1/2 were observed in B/myeloid MPAL, while T/myeloid MPAL exhibited epigenetic modifications in genes associated with T-cell signaling pathways (34).

Consistent with these findings, other experimental studies revealed that phenotypically diverse blast subpopulations in MPAL patients possess similar genetic alterations, thereby supporting the hypothesis that the mixed-lineage phenotype originates from a shared ancestral clone rather than from independent molecular mutations (5, 35, 36).

Epigenetic deregulation also remains a crucial contributor to the pathogenesis and phenotypic plasticity of MPAL. While genetic alterations define the oncogenic drivers of the disease, several studies have demonstrated that aberrant DNA methylation, histone modification, and chromatin remodeling play central roles in sustaining the mixed-lineage transcriptional program.

In one of the earliest studies addressing this concept, Kotrova et al. revealed that unique genetic lesions did not underpin distinct immunophenotypes but rather by epigenetic differences influencing lineage-associated gene expression. These findings suggested that lineage ambiguity can arise through reversible epigenetic reprogramming rather than fixed mutational events (35).

Recently, Mulet-Lázaro et al. provided direct mechanistic insights by integrating multi-omic datasets, including ATAC-seq, ChIP-seq, and methylation profiling, across mixed myeloid/lymphoid leukemias. Their findings revealed epigenetic alterations within hematopoietic regulatory networks—particularly in the CEBPA, PAX5, and GATA1 loci—that modify chromatin accessibility and impair lineage fidelity. Moreover, the study demonstrated that pharmacological inhibition of epigenetic modifiers could partially restore normal lineage programs *in vitro*, highlighting their potential as therapeutic targets (37).

In summary, the presented studies suggest that phenotypic diversity in MPAL reflects mutations acquired in a multipotent progenitor rather than ongoing genomic evolution.

### Genetic abnormalities

#### Cytogenetics

With reference to the recent updates in the classification standards of WHO 2022 and ICC 2022, four distinct subtypes of MPAL are diagnosed based on cytogenetic alterations: MPAL with *BCR::ABL1* and MPAL with *KMT2Ar*, already included in previous classifications, and novel subtypes: MPAL with *ZNF384r* and MPAL with *BCL11B* activation (1, 15). The prevalence of genetic abnormalities varies between MPAL subtypes classified by immunophenotype and age groups.

Cytogenetic abnormalities are present in 64% to 87% of MPAL patients (15). The most frequently reported ones include: complex karyotype, *t*(9;22) and 11q23 rearrangements involving the *KMT2A* gene (4, 34).

The occurrence of complex karyotype and *t*(9;22) correlates positively with advancing age and B/myeloid MPAL, whereas *KMT2Ar* are predominantly identified in pediatric populations, particularly among patients diagnosed with the B/myeloid subtype. The most frequent *KMT2A* patterns are *AFF*, *MLLT3* and *MLLT1* (10, 34).

The *BCR::ABL1* fusion is detected with significantly higher frequency in the adult population compared to the pediatric population (8.2%–23.9% vs. 1.7%–3.9%, respectively) (31–34). There is no consensus about the predominance of the isoform of BCR::ABL1 fusion. Numerous studies present conflicting findings, with some reporting a higher frequency of p190 (31) or p210 (32) isoforms, while others document equal expression levels (33). Although MPAL with *t*(9;22) has been frequently reported to exhibit additional cytogenetic abnormalities, predominantly complex karyotypes, monosomy 7, and trisomy 8, there remains no consensus on the defining cytogenetic abnormalities or their association with adverse outcomes (4, 38, 39).

Nevertheless, chromosomal aberrations have been extensively studied in MPAL with *t*(9;22); however, molecular mutations, by contrast, have been relatively under-investigated. Recently, *RUNX1* mutations were reported to be frequently identified in MPAL with *BCR::ABL1* fusion; nonetheless, their impact on survival prognosis remains uncertain (31, 33–35).

*ZNF384r* is present in approximately 50% of pediatric B/myeloid MPAL cases but is rarely detected in adult cases (27). This MPAL subtype exhibits transcriptional and epigenetic profiles comparable to those of B-ALL with *ZNF384r* (40). In comprehensive research on genetic abnormalities in MPAL, Alexander et al. revealed that B/myeloid MPAL with *ZNF384r* presents leukemia cells at a more mature stage of development than other types of B/myeloid MPAL. It was concluded that *ZNF384r* most likely characterizes acute leukemias that vary immunophenotypically from B/myeloid MPAL to B-ALL and are inclined to harbor FLT3 mutations (34).

Approximately 10%–15% of MPAL cases and 20%–30% of T/myeloid MPAL are characterized by *BCL11Br* (1). These recurrent genetic alterations encompass the entire phenotypic spectrum of acute leukemias. They are identified not only in T/myeloid MPAL but also in approximately one-third of ETP-ALL cases and in a small subset of AML with minimal differentiation or without maturation, thereby highlighting the existence of a biological continuum (41, 42). Along with this statement, MPAL and ETP-ALL are distinguished solely by a single marker, MPO, rather than leukemia-driving genomic aberrations.

Unlike the pathogenic mechanisms described in T-ALL, MPAL with *BCL11B* activation carries 14q32 structural variants that preserve, rather than disrupt, the *BCL11B* coding sequence. These rearrangements promote enhancer hijacking, leading to allele-specific overexpression of *BCL11B* in hematopoietic stem and progenitor cells, where the gene is typically silenced. Such dysregulation of gene expression contributes to lineage aberrancy (43). Further analysis of co-occurring genomic alterations revealed that 80% of MPAL cases with *BCL11Br* harbored *FLT3-ITD* mutations, which may serve as a basis for targeted therapy (41).

### Mutation profile

**Figure 2:**
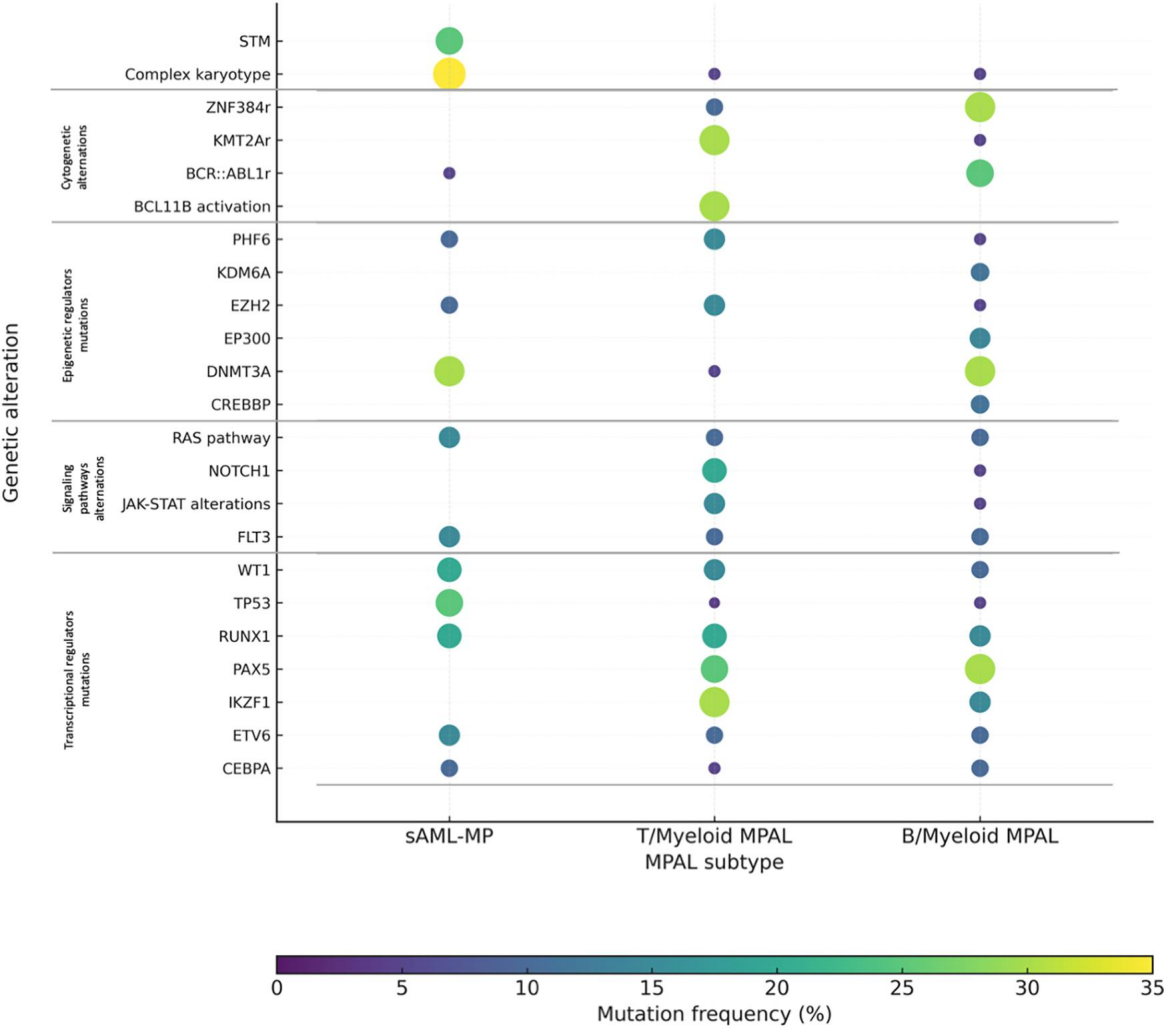
The distribution of genetic alterations across MPAL subtypes.

Mutations identified in MPAL are commonly observed in both AML and ALL, however B/myeloid and T/myeloid MPAL present different mutational patterns (Figure 2). With the advent of next-generation sequencing (NGS), an increasing number of mutations have been described in MPAL patients, including: *NOTCH1* (7%–29%), *RUNX1* (8%–26%), *DNMT3A* (22%–26%), *IDH2* (7%–33%), *ASXL1* (6%–25%), *SRSF2* (5%–23%), *NRAS* (5%–23%), and *TET2* (3%–8%), further underscoring the heterogeneity of the disease (13, 23).

**Figure 3:**
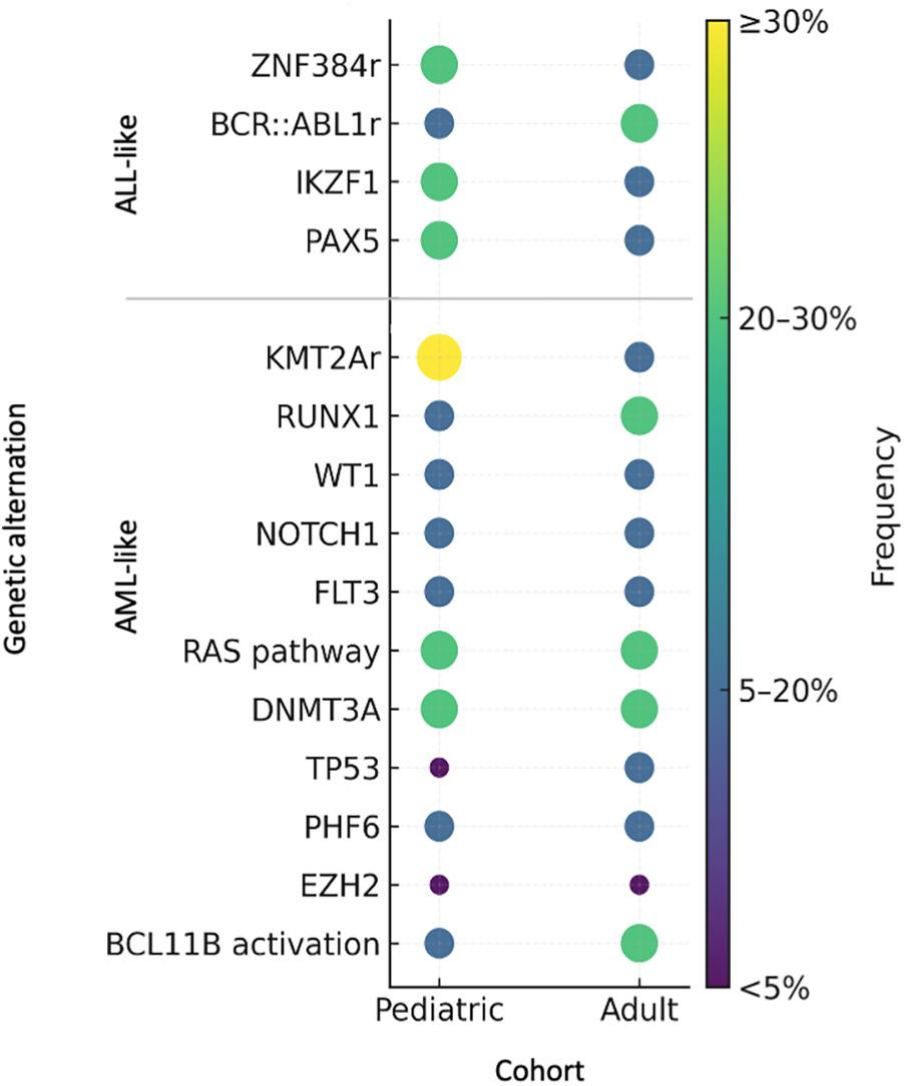
The distribution of genetic alternation across age groups in MPAL.

An extensive pediatric and adolescent study conducted by Alexander et al. demonstrated that T/myeloid MPAL carries a substantial mutational burden, predominantly characterized by alterations in transcriptional regulators such as *WT1*, *ETV6, RUNX1*, and *CEBPA*, which tended not co-occur. These cases also frequently exhibited lesions affecting the *JAK–STAT* pathway, *FLT3* mutations as well as epigenetic modifiers including *EZH2* and *PHF6*. In contrast, B/Myeloid MPAL displayed a lower overall number of genetic abnormalities, most commonly involving *IKZF1, PAX5*, and mutations within the *RAS* signalling cascade, particularly *NRAS* and *PTPN11*. Notably, the mutational landscape of T/Myeloid MPAL demonstrated considerable similarity to that of ETP-ALL. Additional investigations in adult or mixed-age cohorts further associated T/Myeloid MPAL with recurrent mutations in *CEBPA*, *DNMT3A*, *PHF6*, and *NOTCH1*, whereas B/Myeloid MPAL was more frequently linked to alterations in *RUNX1* and components of the *RAS* pathway (23, 44). In adults, the genomic profile shifts toward a higher prevalence of age-associated mutations characteristic of stem/myeloid-biased disease such as *DNMT3A*, *IDH1*, and *IDH2,* aligning with other forms of acute leukemia (45, 46). This distribution reflects greater clonal complexity and is associated with poorer prognosis in adults. The prevalence of gene mutations across different age groups is summarized in Figure 3.

Current WHO and ICC classifications emphasize diagnosis based on genetic mutations, reassigning many cases with myelodysplasia-related cytogenetic or mutational features to AML rather than MPAL. This reclassification is important because several myelodysplastic abnormalities, such as complex karyotype, were previously included in MPAL cohorts, thereby inflating their frequency and obscuring the true disease biology (3). Recent studies applying updated criteria still report a substantial proportion of MPAL with myelodysplasia-related cytogenetics and mutations (e.g., complex karyotype, del5q/–5, del7q/–7, del17p/–17, trisomy 8, and del13q, in ∼28%–32% of adults).

Comparative studies demonstrate that patients with secondary AML showing mixed-phenotype features (sAML-MP) have distinctly poorer outcomes than MPAL. sAML-MP is enriched for myelodysplasia-related genetic abnormalities, exhibits low remission rates with ALL-type induction (14% vs. 97% in MPAL), and shows significantly inferior survival (median OS 10.3 vs. 42.8 months; HR ∼2.3) (36). Multivariable analyses consistently indicate that myelodysplasia-related molecular mutations and complex karyotype, but not immunophenotype, are the dominant biological and prognostic drivers. Consequently, patients with myelodysplastic-defining cytogenetics or myelodysplasia-related molecular mutations should be classified and treated as myelodysplasia-related AML, except for RUNX1-mutated cases, which retain clinical and biological features more consistent with MPAL (36, 47).

Eventually, the pivotal research of Takahashi et al. about epigenetic patterns in MPAL should be presented. They conducted an integrated analysis of DNA methylation and gene expression in 31 MPAL cases, identifying lineage-specific epigenetic programs. Their work showed that T/myeloid MPAL exhibits hypomethylation patterns consistent with T-cell receptor signaling, whereas B/myeloid MPAL displays methylation profiles supporting B-lineage transcriptional activity. Importantly, patients treated with induction regimens matched to their methylation-defined lineage achieved markedly higher complete remission rates (72% vs. 22%; *P* = 0.037), although this strategy did not improve overall survival (23).

In summary, a specific and comprehensive molecular assay in MPAL has yet to be developed. Cytogenetic testing, including BCR::ABL1r, KMT2Ar, ZNF384r, and BCL11B activation, should be performed to ensure accurate diagnosis. Furthermore, the mentioned cytogenetic abnormalities may serve as risk factors (e.g., KMT2Ar) or as targets for specific therapies (e.g., BCR::ABL1, KMT2Ar). Additionally, genetic mutations such as FLT3, JAK, or IDH should be considered, as targeted therapies are available. Eventually, comprehensive molecular assays for NGS testing could be valuable for risk stratification, or, in the future, reclassification and targeted therapies.

### Prognostic factors

Although risk-adapted therapeutic approaches are standard in other acute leukemias, there are insufficient data to support the application of risk-based strategies in MPAL. Limited literature reports suggest that MPAL is associated with a poorer prognosis compared with classical forms of AML or ALL, although findings in pediatric patients remain inconclusive. The outcomes of MPAL therapy are generally better in children and younger adults than in older adults (2–4, 9, 10).

Knowledge concerning biological factors that influence prognosis in MPAL remains quite limited. The negative prognostic significance of *t*(9;22) and KMT2Ar has been demonstrated (2, 5, 13, 14). In the analysis conducted by Matutes et al., the median overall survival (OS) for MPAL patients with a normal karyotype was 139 months, compared with 28 months for KMT2Ar MPAL and 8 months for Ph(+) MPAL (3).

However, most of the reports concerning the inferior prognosis associated with MPAL with *t*(9;22) were published prior to the advent of extensive therapy involving tyrosine kinase inhibitors (TKIs) in all acute leukemia exhibiting *BCR::ABL1r*. In the large research analysis, Qasrawi et al. demonstrated that patients with MPAL harboring *t*(9;22) experienced a decreased risk of mortality and better clinical outcomes compared to other MPAL subtypes (HR = 0.28, [CI, 0.13–0.62], *P* = .002) (48).

Additional adverse prognostic markers include elevated leukocyte counts, extramedullary involvement at the time of diagnosis, failure to achieve remission subsequent to induction therapy, and the type of therapy administered. It is generally documented that a significant survival improvement can be achieved with ALL-like induction followed by allo-HSCT (3, 4, 10, 49). Nonetheless, survival analysis within pediatric populations indicates that allotransplantation does not enhance OS in children who achieve deep remission after induction (22).

Furthermore, prognostic differences appear to exist between bilineal and biphenotypic leukemia. Bilineal MPAL has been associated with poorer prognosis, although due to difficulties in clearly distinguishing it from biphenotypic forms, these categories are not separated in the WHO classification (22, 50).

The expression of low-level MPO in B/myeloid MPAL also appears to have prognostic significance. Cases exhibiting isoMPO expression may have differing prognoses from those of classical MPAL and B-ALL. Available research indicates that adult patients with B/myeloid MPAL with isoMPO experienced significantly better outcomes compared to those with other MPAL subtypes (OS undefined, 16 months, *p* < 0.05) (21). Given the therapeutic implications of these findings, further studies will be essential to elucidate their clinical relevance.

Last, but not least, the role of minimal residual disease (MRD) in risk assessment needs to be discussed. Despite the pivotal role of MRD as a prognostic factor in the treatment guidance of other acute leukemias, its significance in the risk stratification and therapeutic decisions for MPAL remains uncertain. In retrospective analyses, flow-based MRD negativity was significantly associated with improved survival (22, 51, 52).

Oberley et al. demonstrated that, within the pediatric population MRD) positivity was predictive of 5-year event-free survival (5yr-EFS) (HR = 6.00, *p* < 0.001) and OS (HR = 9.57, *p* = 0.003). Furthermore, earlier MRD clearance was associated with improved treatment outcomes and increased survival rates (52).

However, there is a lack of such comprehensive analysis in adults. Therefore, MRD-guided therapy is not applied to this population. Due to data limitations, further investigation into the utility of MRD in MPAL treatment should be conducted extensively. The advent of molecularly assessed MRD may further enhance predictive accuracy.

### Treatment

To date, no standardized therapeutic regimen has been established for MPAL. Current knowledge regarding treatment efficacy is derived exclusively from retrospective analyses, which are limited by heterogeneous patient cohorts and the use of different diagnostic classifications (EGIL, subsequent WHO or ICC classifications).

### Optimal induction chemotherapy in pediatric and adult patients

Most retrospective studies, including mixed, adult and pediatric, cohorts, have demonstrated higher complete remission (CR) rates and longer OS in MPAL patients treated with ALL-like regimens compared with AML-based approaches (2, 3, 10, 53). According to a meta-analysis of 1351 patients diagnosed with either 2008 WHO-defined or EGIL-defined MPAL, AML-like induction chemotherapy was associated with a significantly lower likelihood of achieving CR (odds ratio: 0.33; 95% CI: 0.18–0.58). Additionally, OS was also favored in patients treated with an ALL-like protocol (OR = 0.45, 95% CI 0.26–0.77). However, this effect was not observed in a multivariable analysis that included treatment type, patient age, and MPAL subgroup (10). Nevertheless, most studies restrict their induction reports to AML or ALL-like information, without specifying the administered regimens or classifying different protocols under these two categories, thereby relying on the discretion and experience of each clinical center.

In the pediatric population, the two most comprehensive analyses to date present contradictory outcomes. The International Berlin-Frankfurt-Munster Study (iBFM-AMBI1012) demonstrated that, within a cohort of 233 children and adolescents with MPAL, the ALL-like induction yielded a superior 5yr-EFS compared to AML-like or hybrid treatment modalities (80% ± 4%, 36% ± 7.2%, and 50% ± 12%, respectively) (22). Conversely, Orgel et al. report in the Children's Oncology Group study that patients receiving induction therapy for ALL-like or AML-like conditions exhibited comparable outcomes (54). The treatment recommendation algorithm presented by Hrusak et al. assumes ALL-like treatment in the majority of MPAL cases, with the addition of TKIs in the presence of *BCR::ABL1r*. However, the AML-like protocol is recommended if cytogenetic abnormalities such as *RUNX1::RUNX1T1*, *PML::RARA* or *CBFB::MYH11*are detected or in case blast cells express phenotype: CD19(−), CD3(+), CD7(+) or CD19(−) and at least 2 of CD10, CD79a or CD22 positivity (22).

In turn, recommendations for the treatment of adult MPAL patients are primarily based on retrospective analyses of therapeutic outcomes involving mixed pediatric and adult cohorts (15, 49). Research exclusively targeting adult patients has documented improved CR rate with ALL-like regimens, however, no statistically significant OS advantage was observed with this treatment approach (45, 51).

In the study conducted by Doung et al., the efficacy of the hyperCVAD protocol as a specific treatment was evaluated in a cohort of 25 adult patients. The median age of 49 years, overall response rate (ORR) was 84%, and 66% of the patients who achieved CR/CRi) proceeded to alloHSCT. Median OS was not reached; however, the estimated two-year survival rate was 63% (55). Accordingly to previous studies, young adults (patients under 40) were recommended for treatment with intensive pediatric ALL protocols (3, 4, 10).

Hybrid regimens, with activity against both lymphoid and myeloid blasts, represent an alternative. Although remission rates are similar to those achieved with ALL-based protocols, investigators have noted higher toxicity, likely due to combining intensive 3 + 7 regimens with vincristine and corticosteroids (2, 4, 8). More tolerable hybrid regimens include FLAG-IDA and CLAG-M. In a study by the Polish Adult Leukemia Group (PALG), the overall response rate reached 75%, with toxicity levels comparable to those observed in standard ALL or AML protocols (56, 57). Recent analysis of the PALG and PETHEMA MPAL registries demonstrated that, among 304 adult patients, the CR/CRi rate was notably higher in the CLAG-M/FLAG-IDA group compared to the AML-like and ALL-like groups (75% vs. 58% and 69%; *p* = 0.02) (58).

Regardless of age group, given the increased frequency of CNS involvement at diagnosis, intrathecal triple-agent chemotherapy should be administered to all patients upon diagnosis of MPAL (5–7). Furthermore, in cases of resistance to initial therapy or relapse, a transition to acute myeloid leukemia (AML)-type treatment is recommended. This approach considers the potential for clonal expansion of blasts from a different lineage under selective pressure, such as the development of myeloid clones following ALL-like therapy (15, 22, 49).

Attempts have also been made to tailor treatment by classifying MPAL cases as more “AML-like” or “ALL-like” based on mutation patterns, gene expression profiles (34), or methylation signatures (23). Takahashi et al. demonstrated that patients benefited from regimens matched to their methylation-defined subtype (AML-like or ALL-like). In comparison between patients who received matched therapy and those who received unmatched therapy, the CR rates were 72% and 22%, respectively, *p* = 0.037. However, the improvement in CR rate did not translate into an OS benefit (23).

Recently, an subtype of MPAL with myelodysplastic-related mutations has been extensively evaluated. Research by Galera et al. demonstrated that in adult patients diagnosed with MPAL with myelodysplastic-related mutations, outcomes were superior compared to secondary AML with mixed phenotype when treated with an ALL-like protocol (CR 96.6% vs. 14.3%, *p* = 0.001). The latter, however, exhibited a better response to AML-like induction (36). Conversely, another study focusing on an adult cohort with myelodysplastic-related mutations did not indicate any improvement in OS) or relapse-free survival (RFS) concerning the type of treatment (47).

In summary, extensive prospective studies remain an unmet need in MPAL. Nevertheless, profound genetic sequencing may reveal more coherent subtypes of MPAL that present superior outcomes when treated with tailored therapies.

### Allogeneic hematopoietic stem cell transplantation

MPAL is regarded as a high-risk variant of leukemia characterized by increased rates of resistance and relapse, potentially due to its origin from an early hematopoietic stem cell. According to the SEER registry, the risk of mortality for MPAL patients was elevated by 59% and 26% in comparison to patients with ALL and AML, respectively (9). While intensive induction followed by alloHSCT is considered the most appropriate therapeutic approach in eligible adults, data from the pediatric population does not definitively indicate a benefit from alloHSCT in children with MPAL.

Consolidation with alloHSCT in adult patients is essential for prolonged survival. Findings from retrospective studies demonstrated comparable survival rates in adult MPAL patients who underwent allotransplantation during their first or second remission, to ALL or AML patients also consolidated with alloHSCT (11, 51, 59–62). The most favorable outcomes were observed with myeloablative conditioning regimens (MAC), especially incorporating total body irradiation (TBI) (11, 51, 60).

Furthermore, Liu et al. demonstrated that standard vs. intensified MAC, all protocols based on TBI, in adults <50 y.o. significantly increased 5-years OS (23.8% ± 8.9%, 64.0% ± 8.4% *P* = 0.029) while decreasing the risk of relapse (5-year cumulative incidence of relapse was 80.8 ± 8.5% and 28.8 ± 9.9%, *P* < 0.001) (63).

In the most extensive retrospective analysis conducted to date, Munker et al. evaluated 519 adult patients with MPAL who underwent alloHSCT) during their first remission. The rates of leukemia-free survival (LFS) and OS) were 46.5% (95% CI, 41.7–51.4) and 56.3% (95% CI, 51.5–61.2), respectively. The cumulative incidence of relapse within 3-years following alloHSCT was 31.4% (95% CI, 26.9–35.9).

According to the presented studies, it is generally advisable to proceed with alloHSCT in eligible adult patients who have achieved remission and a suitable donor is available.

While pretransplant MRD status is extensively examined as a prognostic indicator to guide subsequent therapy in AML and particularly in ALL, its impact on allotransplantation outcomes within MPAL patients remains unestablished. The study published by Lazzarotto et al. revealed OS) was superior in patients who were MRD-negative before alloHSCT (75.8% vs. 45.2%, *P* = 0.06) (51). On the contrary, a study by Getta et al. did not demonstrate an effect of MRD on post-transplant survival (60). The role of MRD status in guiding transplant decisions among adults with MPAL remains uncertain, and additional prospective studies are needed to define its prognostic and therapeutic relevance in this population.

Contrary to recommendations for adult MPAL, alloHSCT offers no definitive advantage for the pediatric MPAL population, as the majority can be successfully treated with ALL-based chemotherapy alone (6, 7, 11, 64, 65). Orgel et al. reported that children with early favorable responses to ALL induction who continued with ALL chemotherapy without HSCT in first complete remission experienced a 5-year OS rate exceeding 80% (54).

In pediatrics, alloHSCT is typically considered for patients with induction failure, positive MRD (>5%) at the end of induction or consolidation, or after a lineage switch (54). As in the adult population, TBI is recommended as the basis for conditioning, thereby improving survival (11).

Nevertheless, in both adult and pediatric populations, prospective trials are necessary to clearly establish the subset of patients who benefit from alloHSCT as well as the role of pre-transplant MRD as a prognostic factor for alloHSCT consolidation and OS.

### Targeted therapy

Analogous to Ph + ALL, the incorporation TKIs into treatment regimens has improved outcomes in Ph + MPAL (12, 48, 66). In a retrospective study, Shimizu et al. compared outcomes of 13 patients (age range 16–75, median 53) with Ph + MPAL (*n* = 47) and 27 patients with Ph + ALL treated with imatinib, showing comparable results between the two groups (CR: 100% vs. 85%; 5-year OS: 55% vs. 53%; DFS: 46% vs. 42%) (12). These results were validated in a later comprehensive report involving 241 patients with MPAL, among whom 38 were classified as Ph(+). Within the mixed pediatric and adult cohort, the 2-year OS in Ph(+) patients was 72%, compared to the 2-year OS in Ph(−) patients (median NR vs. 20 months, respectively; HR = 0.45 (CI] 0.27 to 0.75), *p* = 0.002) (48). In conclusion, the management guidelines for both adult and pediatric MPAL recommend the use of TKIs in the treatment of MPAL with *BCR::ABL1r* (15, 22).

As in ALL treatment protocols, maintenance therapy with TKIs should also be considered in Ph + MPAL. Andrews et al. recommend monitoring BCR::ABL1 transcript levels every three months and switching from imatinib to dasatinib or nilotinib if the reduction in BCR::ABL1 levels is less than 4-log at month three, after confirming treatment adherence and excluding the presence of the T315I mutation (13).

The progressive characterization of genetic alterations and more availability in clinical trials have opened new opportunities for the development of targeted therapies in MPAL (Table 4).

**Table 4:** Potential targeted and biological therapies for MPAL.

Therapeutic Approach	Rationale and Examples	Agents	Targeted population (age range)	Ongoing clinical trials ID
Tyrosine Kinase Inhibitors	Applied in BCR-ABL1-positive MPAL. Targeted inhibition of aberrant kinase signaling pathways can improve disease control.	Imatinib	1–21	NCT03007147
		Dasatinib + Venetoclax	>18	NCT04872790
			1–18	NCT06390319
FLT3 Inhibitors	Indicated for MPAL cases harboring FLT3 mutations (particularly T/myeloid MPAL) or with FLT3 pathway overexpression (e.g., B/myeloid MPAL with ZNF384 rearrangement, MPAL with KMT2A rearrangement).			No ongoing trails
T-Cell Engaging Therapies	Blinatumomab and CD19-directed CAR-T cells may be considered for relapsed MPAL expressing CD19, based on extrapolated data from BCP-ALL. Note: treatment line modifications may be necessary.	Blinatumomab + Venetoclax	>18	NCT07222579, NCT04827745
			>14	NCT06991920
			<1	NCT05327894
				NCT06317662
		Blinatumomab + Nivolumab	>16	NCT02879695
		CAR-T cells therapy	18–74	NCT06325748
Phenotype-Directed Therapy	Monoclonal antibodies targeting specific MPAL surface antigens (e.g., CD19, CD20, CD22, CD38, CD123), informed by studies in AML and ALL.	Inotuzumab Ozogamicin	1–25	NCT03959085
		Tagraxofusp	1–21	NCT05476770
			>18	NCT06034470
DOT1L, Menin, or Bromodomain Inhibitors	Inhibition of critical epigenetic regulators and signaling pathways to disrupt leukemogenic mechanisms.	Revumenib	>30 days-old	NCT04065399
			1–6	NCT05761171
		+ Venetoclax	1–30	NCT06177067
			>2	NCT06575296
		Ziftomenib	>18	NCT04067336
		Uproleselan	>17	NCT05146739
		Enasidenib	>18	NCT03683433
Signaling Pathway Inhibitors	Includes BCL2 inhibitors, informed by efficacy in related stem-cell-derived leukemias such as ETP-ALLand, based on AML studies.	Venetoclax	1–18	NCT06390319
			2–30	NCT04898894
			1–40	NCT05292664
			>18	NCT03404193
				NCT04797767
				NCT05901974
				NCT04128501
				NCT02115295

In recent years, several novel agents have been introduced into the treatment of acute leukemia, including small-molecule inhibitors of FLT3, IDH, and BCL2 for AML, as well as CD19, CD22, or CD123 targeted therapies. The summary of clinical trials involving the MPAL population can be found in Table 4.

Venetoclax (Ven), a BCL2 inhibitor, represents one of the most promising and extensively examined novel drugs. Demonstrating its efficacy in AML therapy (67, 68) and in preclinical trials in ALL, including ETP-ALL (69–71), its application in MPAL has garnered significant interest. In reported case series involving adult patients, chemotherapy based on the FLAG-IDA protocol (fludarabine, cytarabine, idarubicin, and filgastrim), supplemented with Ven, has resulted in achieving sustained remission. In a study presented by Ségot et al., all patients were reported with CR with MRD negativity (72). Additionally, another report indicated that two of four MPAL patients treated with Ven-FLAG-IDA as either first-line or salvage therapy achieved CR (73).

Furthermore, the management of aggressive leukemias such as MPAL has been particularly challenging, concerning populations ineligible for intensive treatment or experiencing relapse. Several case reports have demonstrated promising results of therapy with Ven and azacitidine (Aza) or low-dose cytarabine (LDAC) in such patients (74–77).

Eventually, an alternative therapeutic option for patients might be a combination of Ven, Aza, LDAC, and cladribine. As reported in previous studies, cladribine demonstrates effective activity against MPAL in nucleoside analogue-based regimens (56, 57). It is anticipated that the clinical experiences and findings from ongoing trials assessing the Ven-Aza-LDAC-cladribine protocol in unfit AML patients (NCT03586609) may likewise be applicable to unfit MPAL patients.

Unlike the widespread use of venetoclax in adults, clinical experience with this regimen in children remains limited. However, inspired by its promising activity across various hematologic malignancies in adults, venetoclax has been thoroughly studied in the pediatric population (78). Notwithstanding, numerous clinical trials involving ALL and AML, for the MPAL population, a phase I clinical trial is currently recruiting pediatric and young adult patients (ages 1–39) with relapsed MPAL to evaluate the combination of the BCL2 inhibitor venetoclax and CPX-351 (NCT03826992) (79).

Given the sensitivity of *KMT2Ar* acute leukemias to menin inhibition, extending this approach to the *KMT2Ar* subset of MPAL is biologically compelling. Notably, the phase 2 AUGMENT-101 trial that resulted to revumenib approval included only one patient with MPAL, yet this individual achieved morphologic remission (80). In the future, additional research may facilitate the utilization of menin inhibitors in patients with MPAL with *KMT2Ar*.

Evidence suggests that the T/myeloid phenotype is often associated with *FLT3* mutations, and specific MPAL subtypes, such as B/myeloid MPAL with *ZNF384r* or *KMT2Ar*, show activation of the *FLT3* pathway even without somatic *FLT3* mutations. These patients could potentially benefit from *FLT3* inhibitor therapy, although there is currently no clinical trial data to support this approach (13, 81).

Alterations activating the JAK–STAT pathway occur in roughly 10%–20% of Ph-negative ALL, most often through JAK1/2, IL7R, or CRLF2 lesions characteristic of Ph-like disease (82). Preclinical data support the activity of JAK inhibitors in these settings, and several trials are evaluating their use in combination with chemotherapy (83, 84). Whether JAK inhibition will provide therapeutic benefit in ALAL subtypes with JAK–STAT upregulation, such as T/Myeloid MPAL or BCL11B-activated ALAL, remains unknown.

T-cell–engaging therapies targeting CD19 + leukemic blasts, such as blinatumomab and CAR-T cells, are increasingly being used as therapeutic options for patients with B/myeloid MPAL. Currently, information regarding the efficacy and toxicity of CAR-T therapy in MPAL is derived from individual case reports (85–87).

Nevertheless, the results from the phase I clinical trial may offer valuable insights. In a cohort of four adult CD7-positive MPAL patients, of whom 3 experienced relapse after alloHSCT, three out of 4 achieved CR), while one patient did not respond to treatment. Cytokine release syndrome was diagnosed in all cases; however, no occurrences of neurotoxicity were observed. At last follow-up, all patients who achieved CR remained alive (88).

Concerning the study, CAR-T therapy presents an opportunity to achieve CR) for MPAL). However, further research involving larger cohorts and extended follow-up periods is necessary to more comprehensively assess the efficacy of CAR-T therapy.

Several case reports demonstrated promising results in adult MPAL patients treated with blinatumomab, also in combination with Ven (89–92). In case study by Azevedo et al. four patients received blinatumomab combined with Ven, LDAC and cladribine. Although all patients achieved CR MRD negative, one patient relapsed with AML (93).

This case raises a concern regarding targeted therapy in MPAL: the risk of selective pressure on a specific lineage, potentially driving clonal expansion in other blast populations. Most reported lineage switch cases (defined as a change in cellular lineage during therapy or at relapse, predominantly involve transformation from ALL to AML subsequent to targeted therapy and are correlated with poor outcomes (94, 95).

Among B-ALL variants, specific genetic mutations that predispose to myeloid or monocytic antigen acquisition were identified. Cases with *DUX4r,* CRLF2r*, PAX5-P80R* mutations, or *ZNF384* fusions may exhibit an early monocytic shift under therapy, characterized by emergent CD14, CD64, or other myeloid-associated markers (96–98).

The research by Iacobucci et al. identified risk factors for lineage switch as pediatric patients, the use of CD19-T-cell engaging therapies, *EZH2* mutations and *KMT2Ar* (96).

The latest report from the EVOLVE project analyzed 70 cases involving both adults and children who experienced lineage switching subsequent to immunotherapies, specifically CAR-T cell therapy (48.6%) and blinatumomab (44.3%). In most cases *KMT2Ar* (*n* = 45, 64.3%) was detected. The majority of patients (75.7%) transitioned from BCP-ALL to AML, while the remaining cases involved transitions from BCP-ALL to B/Myeloid MPAL or AUL. The lineage switch manifested within six months of the most recent immunotherapy in 57 patients (81.4%). Treatment outcomes were poor. Only 20 of 65 patients (30.8%) achieved CR following first-line therapy and OS was 4.8 months.

Collectively, these studies highlight the challenges of managing MPAL with novel targeted agents. A deeper understanding of the biology underlying MPAL evolution and lineage switch mechanisms may help to optimize the use, combination, and sequencing of targeted therapies in this setting.

### Summary

MPAL continues to pose significant diagnostic and therapeutic challenges. Improved understanding of genetic abnormalities characteristic of MPAL will refine diagnostic criteria and enhance outcomes through the development of targeted therapies.

Given the rarity and heterogeneity of MPAL, large multicenter prospective studies are urgently needed in this patient population.
